# Mutational Analysis of a Wheat *O*-methyltransferase Involved in Flavonoid Metabolism

**DOI:** 10.3390/plants11020164

**Published:** 2022-01-08

**Authors:** Alexander B. Cain, Shu Yu, Li Tian

**Affiliations:** 1 Department of Plant Sciences, University of California, Davis, CA 95616, USA; abcain@ucdavis.edu (A.B.C.); shuyu@ucdavis.edu (S.Y.); 2 Nobell Foods, 6000 Shoreline Court, Suite 304, San Francisco, CA 94080, USA

**Keywords:** flavone, *O*-methyltransferase, OMT1, OMT2, tricin, wheat

## Abstract

Methylated flavones, and tricin in particular, have been implicated in protecting wheat plants against a variety of biotic and abiotic stresses. Methylated flavones are produced via *O*-methylation of the hydroxyl groups in flavones, which is catalyzed by *O*-methyltransferases (OMTs). To examine the role of wheat OMT2 in methylated flavone biosynthesis and facilitate interrogation of tricin functions in wheat-environment interactions, loss-of-function mutants of *OMT2* homoeologs, *omt-A2* and *omt-B2*, were identified from a tetraploid wheat Targeting Induced Local Lesions in Genomes (TILLING) mutant population and crossed to generate the *omt-A2* *omt-B2* double mutant. Although tricin and most other soluble phenolics did not differ in leaves and glumes of TILLING control and the *omt-A2*, *omt-B2*, and *omt-A2 omt-B2* mutants, chlorogenic acid was increased in glumes of *omt-A2 omt-B2* relative to TILLING control, suggesting that it might serve as a substrate for OMT2. The *omt2* mutant lines showed similar growth phenotypes as well as comparable lignin deposition in cell walls of stems compared to TILLING control. These results collectively suggest that OMT2 and its close homolog OMT1 may possess overlapping activities in tricin production, with OMT1 compensating for the missing OMT2 activities in the *omt2* mutant lines.

## 1. Introduction

Flavones are a class of phenolic specialized metabolites that are produced to help plants endure a wide range of biotic and abiotic stresses [1]. Methylated flavones, and tricin (5,7,4′-trihydroxy-3′,5′-dimethoxyflavone) in particular, have been implicated in protecting against fungal growth [2,3,4], ultraviolet radiation [5], and cold-temperature stress [6]. Tricin has also been identified as a component of lignin in wheat [7], maize [8], and giant reed (*Arundo donax*) [9] and was suggested to serve as a nucleation site for lignin polymerization in monocots [10].

**Figure 1:**
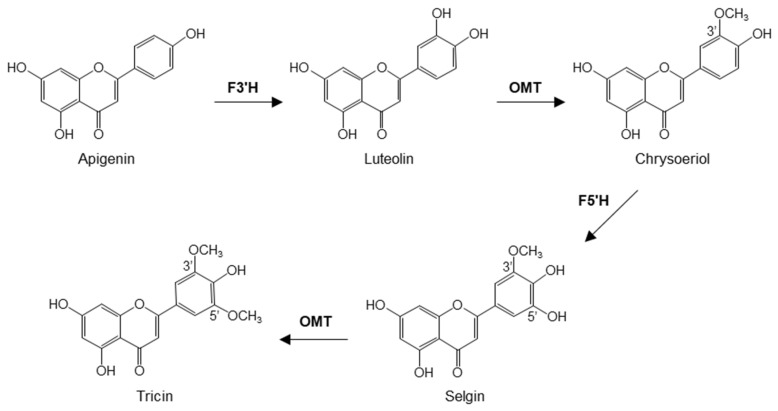
Biosynthesis of tricin from apigenin. F3′H, flavonoid 3′-hydroxylase; OMT, *O*-methyltransferase; F5′H, flavonoid 5′-hydroxylase.

Several enzymatic steps are employed for the conversion of apigenin (5,7,4′-trihydroxyflavone) into tricin, according to studies in rice [11] (Figure 1). Apigenin is hydroxylated at the 3′ position to form luteolin (5,7,3′4′-tetrahydroxyflavone), which is then methylated by an *O*-methyltransferase (OMT) to produce chrysoeriol (5,7,4′-trihydroxy-3′-methoxyflavone). The hydroxylation of chrysoeriol at the 5′ position gives rise to selgin (5,7,4′,5′-tetrahydroxy-3′-methoxyflavone). The final step of tricin biosynthesis is its *O*-methylation by an OMT, performing the 5′-*O*-methylation of selgin to tricin [11] (Figure 1). The OMTs for tricin biosynthesis belong to the broader class of caffeic acid 3-*O*-methyltransferase (COMT) family of OMTs that transfer a methyl group from *S*-adenosyl-L-methionine (SAM) to hydroxyl groups of diverse substrate structures [12].

Wheat is one of the major staple crops. It supplies over 730 million metric tons of grain annually, but its production is challenged around the world by a variety of environmental stresses such as extreme temperatures, drought, and fungal diseases. In view of the defense functions demonstrated by tricin in other plant species, understanding the biosynthesis and role of tricin in wheat and its interactions with the environment can potentially improve wheat growth and yield. Both the 3′- and 5′-*O*-methylation reactions in tricin biosynthesis were proposed to be carried out by the enzyme OMT2 in wheat [13]. In in vitro reactions, wheat OMT2 catalyzed the stepwise 3′-, 5′-, then 4′-*O*-methylation of tricetin (5,7,3′,4′,5′-pentahydroxyflavone) to selgin, then tricin, and finally 3′,4′,5′-*O*-trimethyltricetin, respectively, utilizing SAM as a methyl donor [13,14]. OMT2 had the highest in vitro activity on tricetin and was highly active towards luteolin and 5-hydroxyferulic acid [13]. However, the function of wheat OMT2 has not been investigated in planta.

To understand the in planta activity of OMT2 and examine the role of tricin in wheat growth and environmental interactions, mutants of *OMT2* homoeologs (*omt-A2* and *omt-B2*) were identified from a tetraploid wheat Targeting Induced Local Lesions in Genomes (TILLING) mutant population. The *omt-A2 omt-B2* double mutant was also generated through crossing *omt-A2* and *omt-B2* and genotyping the segregating progenies. The single and double mutants of *omt-A2* and *omt-B2*, along with the control plants, were evaluated for growth and biochemical phenotypes.

## 2. Results

### 2.1. TILLING Mutants with Premature Stop Codons in OMT-A2 and OMT-B2 Were Isolated and the Double Mutant of omt-A2 omt-B2 Was Generated

**Figure 2:**
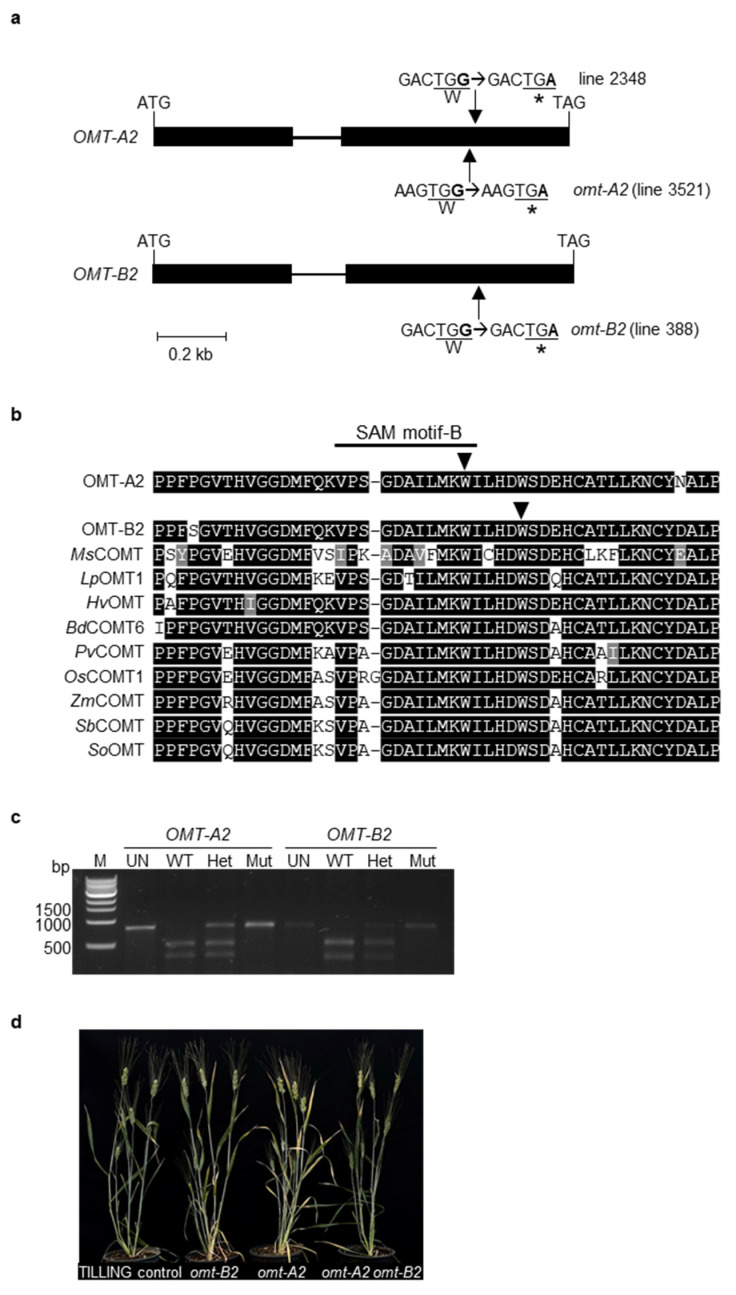
Isolation and molecular characterization of TILLING mutants of wheat *O*-methyltransferase 2 (OMT2) homoeologs. (**a**) Intron-exon organization of *OMT-A2* and *OMT-B2*. Boxes and lines signify exons and introns, respectively. Arrows indicate sites of premature stop-codon mutations. (**b**) Protein sequence alignment of selected plant OMTs. The arrows indicate the sites of stop codon mutations in *OMT-A2* and *OMT-B2*. The GenBank accession numbers of the plant OMTs are: *Bd*COMT6, XP_003573470.1; *Hv*OMT, ABQ58825.1; *Lp*OMT1, AAD10253.1; *Ms*COMT, P28002.1; *Os*COMT1, XP_015650053.1; *Pv*COMT, ADX98508.1; *Sb*COMT, ADW65743.1; *So*OMT, O82054.1; *Zm*COMT, Q06509.1. *Bd*, *Brachypodium distachyon*; *Hv*, *Hordeum vulgare*; *Lp*, *Lolium perenne*; *Ms*, *Medicago sativa*; *Os*, *Oryza sativa*; *Pv*, *Panicum virgatum*; *Sb*, *Sorghum bicolor*; *So*, *Saccharum officinarum*; *Zm*, *Zea mays*. (**c**) Cleaved amplified polymorphic sequence (CAPS) markers for *OMT-A2* and *OMT-B2*. The PCR products were either undigested (UN) or incubated with the respective restriction enzyme and then separated on a 1% agarose gel. M, molecular marker; WT, wild-type plant; Het, heterozygous mutant plant; Mut, homozygous mutant plant; bp, base pair. (**d**) Three-month-old TILLING control and mutant plants.

To identify *omt-A2* and *omt-B2* mutants, the tetraploid wheat cv. Kronos TILLING mutant database [15] was searched using *OMT-A2* and *OMT-B2* sequences from hexaploid wheat cv. Chinese Spring as a query. A total of 73 high-confidence (minimum coverage of 5 or more reads) mutations were identified within the *OMT-A2* open reading frame (ORF) that include 27 synonymous substitutions and 46 non-synonymous substitutions (data not shown). Of the non-synonymous substitutions, 44 are missense mutations, and 2 are nonsense mutations that result in premature stop codons in *OMT-A2* (lines 3521 and 2348) (Figure 2a). Within the ORF of *OMT-B2*, 52 high-confidence mutations were identified that include 17 synonymous substitutions and 35 non-synonymous substitutions (data not shown). Of the non-synonymous substitutions, one caused a premature stop codon in *OMT-B2* (line 388) (Figure 2a).

The premature stop codons in *OMT-A2* (mutant line 3521; designated *omt-A2*) and *OMT-B2* (line 388; designated *omt-B2*) lead to truncated proteins that lack 98 and 93 amino acids from the C-terminal end, respectively (Figure 2a). These missing amino acids are located within a highly conserved region among plant OMTs and encompass one of the previously reported SAM binding motifs (Figure 2b) [16]. As such, the resultant truncated OMT2 proteins do not possess the amino acids critical for enzyme activity and likely lead to loss-of-function mutants.

To generate the *omt-A2 omt-B2* double mutant with knockouts in both OMT-A2 and OMT-B2 activities, *omt-A2* was backcrossed with the parental line Kronos to reduce the level of background mutations; the homozygous *omt-A2* mutant (BC_1_F_2_) was then crossed with the homozygous *omt-B2* mutant (M_4_). Homozygous wild type (*OMT-A2 OMT-B2*; designated TILLING control), single mutant (*omt-A2*, *omt-B2*), and double mutant (*omt-A2 omt-B2*) plants were identified from the segregating progenies using cleaved amplified polymorphic sequence (CAPS) markers (Figure 2c) and subjected to further analysis.

### 2.2. The omt2 Mutants Showed Similar Growth Phenotypes to TILLING Control

**Figure 3:**
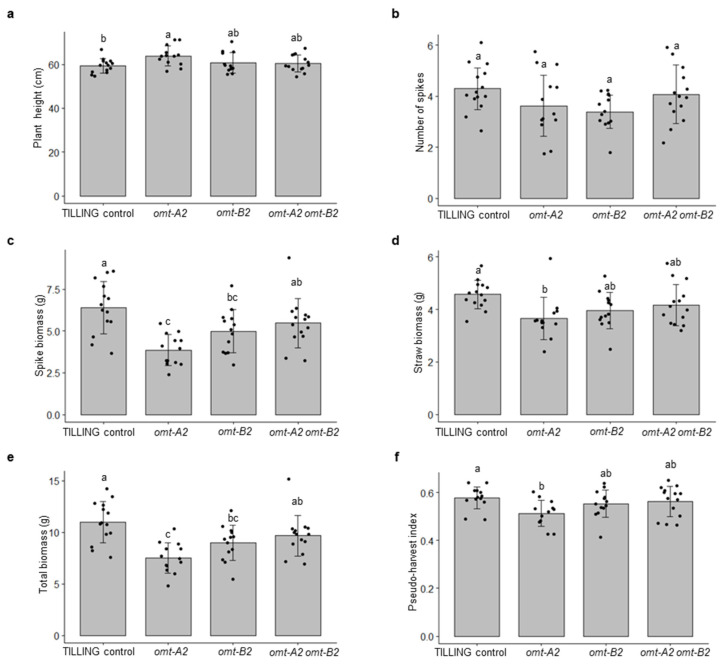
Growth data of TILLING control and mutant plants of wheat *O*-methyltransferase 2 (OMT2) homoeologs. Means ± S.D. of TILING control (n = 14), *omt-A2* (n = 13), *omt-B2* (n = 13), and *omt-A2 omt-B2* (n = 14) for plant height (**a**), number of spikes (**b**), spike biomass (**c**), straw biomass (**d**), total biomass (**e**), and pseudo-harvest index (**f**) are shown. Different letters indicate significant differences (*p* < 0.05) between genotypes for each growth parameter.

TILLING control and the *omt2* mutant plants did not display obvious differences in their growth and development (Figure 2d). To further assess the growth parameters of these plants, the height, number of spikes, straw biomass, spike biomass, total biomass, and pseudo-harvest index of the mature *omt-A2*, *omt-B2*, and *omt-A2 omt-B2* mutants and TILLING control plants were determined and compared (Figure 3). The height for all mutant lines tended to be slightly greater than that of TILLING control, with *omt-A2* plants being significantly taller (*p* < 0.05) (Figure 3a). All of the *omt2* mutants possessed a comparable number of spikes relative to TILLING control (Figure 3b), though the spike biomass of the *omt-A2* and *omt-B2* single mutants was lower than that of TILLING control and the *omt-A2 omt-B2* double mutant (Figure 3c). The *omt2* mutant lines also had a trend of lower straw biomass compared to TILLING control, with the straw of *omt-A2* mutant lines being significantly lighter (*p* < 0.05) (Figure 3d). Additionally, the total biomass of *omt-A2* and *omt-B2* was lower than TILLING control and *omt-A2 omt-B2* (Figure 3e). Furthermore, *omt-A2* mutants displayed a significantly reduced (*p* < 0.05) pseudo-harvest index (mass of spike over total biomass) relative to TILLING control (Figure 3f).

### 2.3. OMT2 Homoeologs Showed Relatively Higher Expression Levels in Reproductive Tissues and the Peduncle

**Figure 4:**
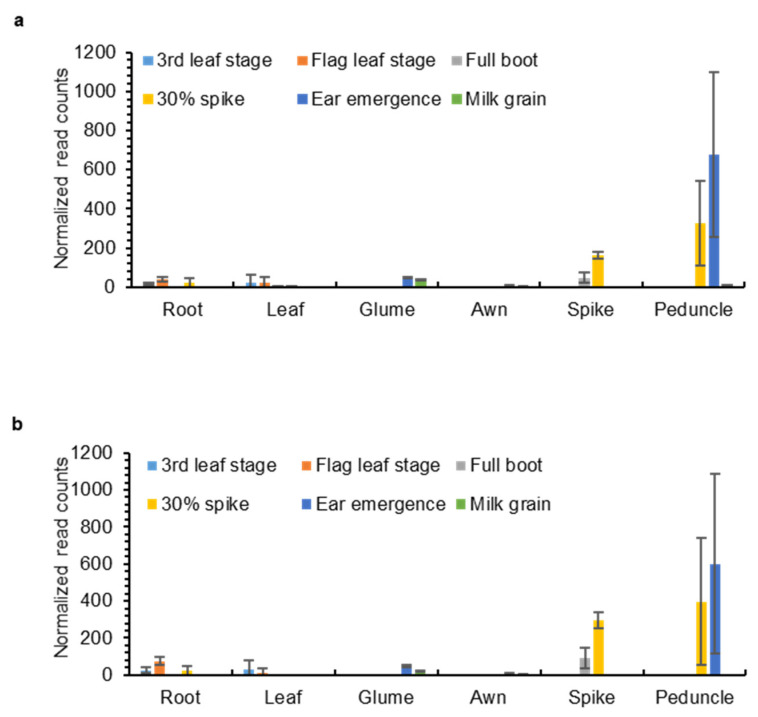
Expression levels of *OMT-A2* (**a**) and *OMT-B2* (**b**) in different wheat tissues. Data were obtained from www.wheat-expression.com (accessed on 18 November 2021) and graphed based on cv. Azhurnaya. Normalized read counts (means ± S.D. of three biological replicates) in roots, leaves, glumes, awns, spikes, and peduncles at 3rd leaf, flag leaf, full boot, 30% spike, ear emergence, and milk grain stages are shown.

To understand the expression and potential function of the *OMT-A2* and *OMT-B2* homoeologs in different wheat tissues, transcript levels of *OMT-A2* and *OMT-B2* were obtained from the Wheat Expression Browser (www.wheat-expression.com; accessed on 18 November 2021), which contains comprehensive transcriptome data across multiple wheat cultivars and tissues [17]. *OMT-A2* and *OMT-B2* exhibited similar expression profiles in various tissues at different developmental stages (Figure 4). While low levels of *OMT-A2* and *OMT-B2* expression were observed for leaves, roots, awns, and glumes at all developmental stages, higher amounts of *OMT-A2* and *OMT-B2* transcripts were present in the spike, particularly when the spike begins to emerge (Figure 4). Besides the reproductive tissues, *OMT-A2* and *OMT-B2* showed the highest expression levels in the peduncle at the 30% spike and ear emergence stages (Figure 4).

### 2.4. Chlorogenic Acid Showed Increased Accumulation in Leaves of omt-A2 omt-B2 Whereas Other Phenolic Metabolites Did Not Measurably Differ

**Figure 5:**
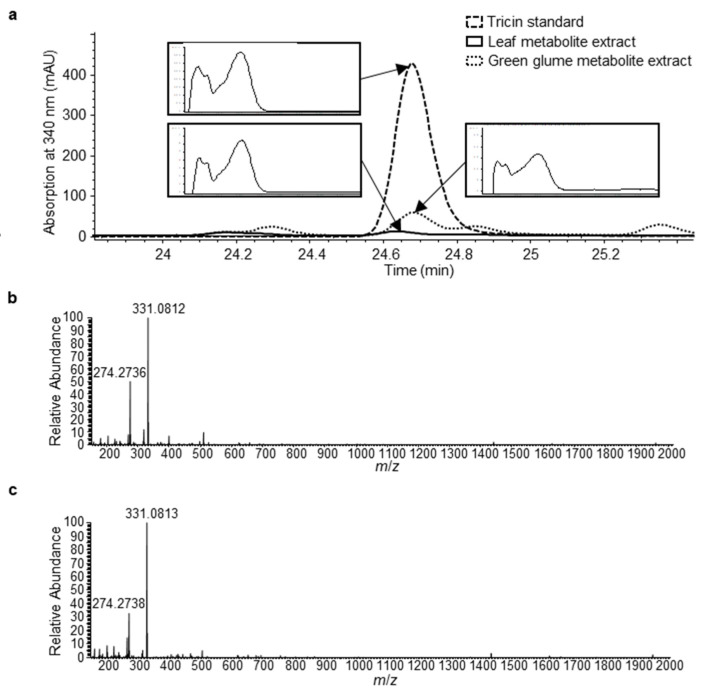
Identification of tricin in leaf and green glume tissues of tetraploid wheat cv. Kronos. (**a**) HPLC chromatograms (absorption at 340 nm) of tricin standard, leaf extract, and green glume extract. Absorption spectra of the indicted peaks are shown in inserts. (**b**) Mass spectrum of the tricin authentic standard. (**c**) Mass spectrum of the HPLC peak from green glume metabolite extract (indicated in panel (**a**)).

To understand whether tricin is produced in leaves and glumes where the *OMT2* homoeologs showed relatively low expression (Figure 4), the glume tissue together with leaves was examined for the accumulation of tricin using high-performance liquid chromatography (HPLC) (Figure 5). The peak eluted at 24.6 min of leaf and glume phenolic metabolite extracts matches the retention time and absorption spectrum of the authentic tricin standard (Figure 5a), and it appears to be more abundant in glumes than leaves. The 24.6 min peak was collected from multiple HPLC runs of glume extracts, pooled, and subjected to mass spectrometry (MS) analysis, which showed a mass spectrum that matches the tricin standard (Figure 5b,c).

**Figure 6:**
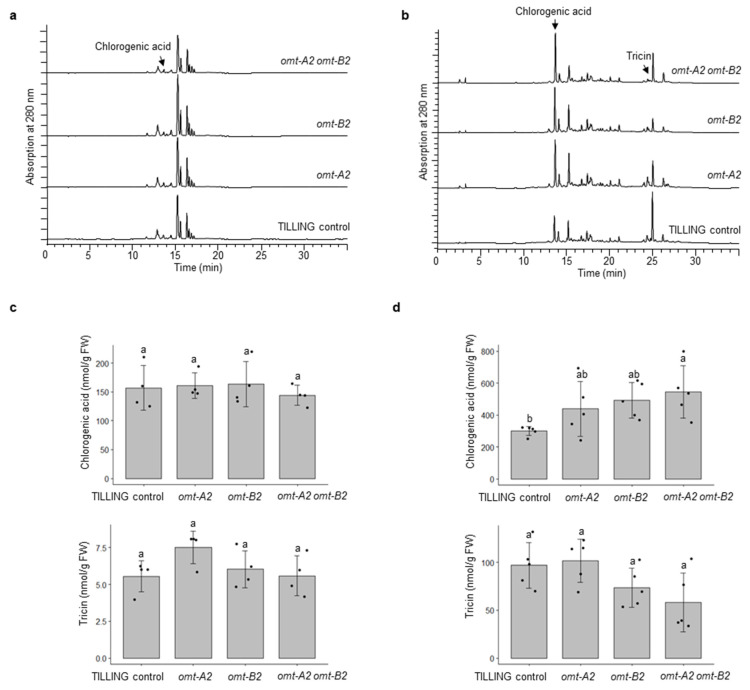
Phenolic profiling of TILLING control and mutant plants. HPLC chromatograms of TILLING control, and the *omt-A2*, *omt-B2*, and *omt-A2 omt-B2* mutants in 4-week-old leaves (4 biological replicates) (**a**), and 8-week-old glumes (5 biological replicates) (**b**) are shown. The peaks corresponding to chlorogenic acid and tricin were quantified and compared among the genotypes for leaves (**c**) and glumes (**d**). Different letters indicate significant differences (*p* < 0.05) between genotypes for each metabolite. FW, fresh weight.

To investigate the effect of *omt2* mutations on phenolic metabolite profiles, leaves from four 4-week-old plants and glumes from 8-week-old plants were collected and processed for metabolite extraction and HPLC analysis. Most of the phenolic metabolites, including tricin, did not show significant changes in their accumulation when either or both *OMT-A2* and *OMT-B2* homoeologs are knocked out (Figure 6). However, a significantly greater accumulation of chlorogenic acid (3-caffeoyl quinate; eluting at 12.5 min) was displayed in glumes of *omt-A2 omt-B2* relative to TILLING control (Figure 6).

### 2.5. The omt2 Mutants and TILLING Control Did Not Differ in Lignin Deposition in the Cell Walls of Wheat Stems

**Figure 7:**
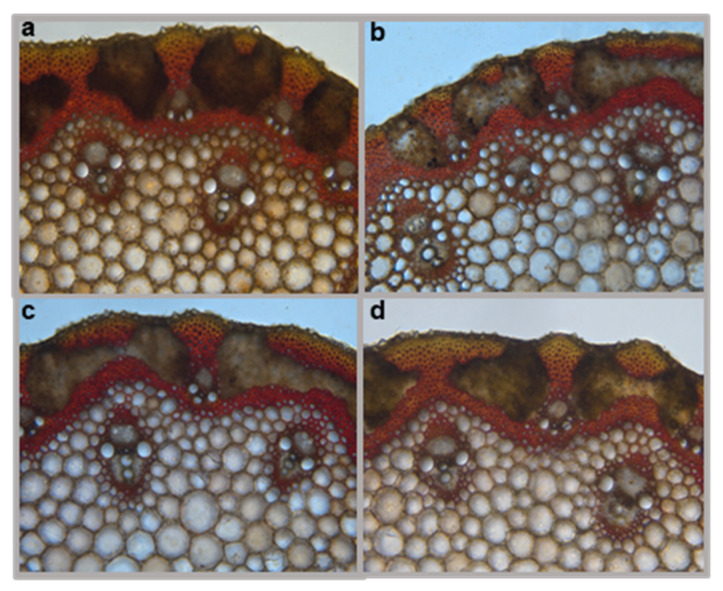
Histochemical analysis of lignin in cross-sections of wheat stems. Representative Mäule staining images of (**a**) TILLING control, (**b**) *omt-B2* mutant, (**c**) *omt-A2* mutant, and (**d**) *omt-A2 omt-B2* mutant are shown.

To assess the effect of *omt-A2*, *omt-B2*, and *omt-A2 omt-B2* mutations on lignin deposition in cell walls, Mäule staining was performed on these mutants and TILLING control under light microscopy (Figure 7). The Mäule staining detected both sinapyl alcohol monomers of lignins that stained red and coniferyl and coumaroyl alcohol monomers of lignins that stained brown (Figure 7). Four stem sections from multiple plants of each of the genotypes were collected, prepared, and stained. Three technical replicates were observed from each plant to assess lignin deposition. Overall, no qualitative differences in lignin deposition were identified among these genotypes (representative images are shown in Figure 7).

## 3. Discussion

Although the enzymatic activity of OMT2 was previously characterized using in vitro assays, this is the first study that explores the function of OMT2 homoeologs in tetraploid wheat employing a genetic approach. The mutated OMT2 proteins are truncated in (OMT-A2) and around (OMT-B2) a SAM binding domain located in a highly conserved region of OMTs, thus are expected to lead to loss-of-function mutants. The *omt-A2 omt-B2* double mutant was generated that abolished the activities of both OMT2 homoeologs and allows the assessment of OMT2 function in tetraploid wheat.

**Figure 8:**
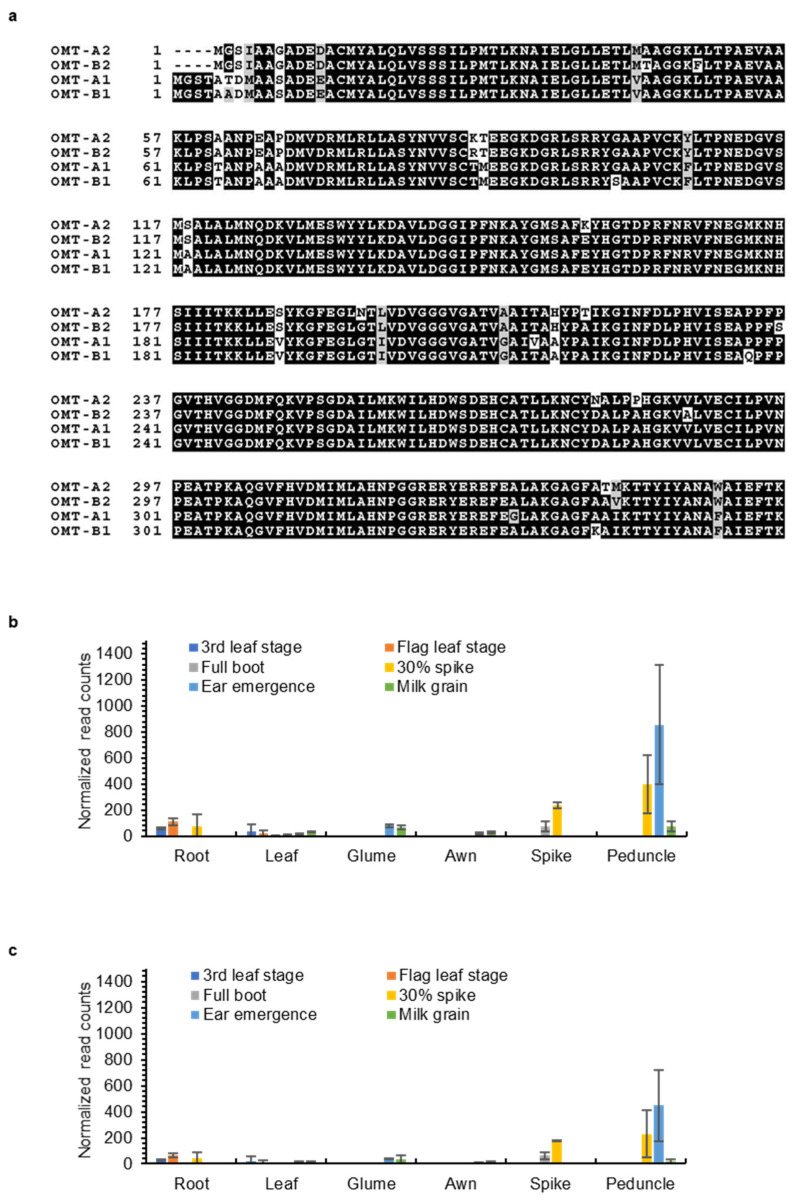
Sequences and expression of wheat *OMT-A1* and *OMT-B1* homoeologs. (**a**) Protein sequence alignment of wheat OMT1 and OMT2 homoeologs. Expression levels of *OMT-A1* (**b**) and *OMT-B1* (**c**) in different wheat tissues. Data were obtained from www.wheat-expression.com (accessed on 18 November 2021) and graphed based on cv. Azhurnaya. Normalized transcript counts (means ± S.D. of three biological replicates) in roots, leaves, glumes, awns, spikes, and peduncles at 3rd leaf, flag leaf, full boot, 30% spike, ear emergence, and milk grain stages are shown.

**Figure 9:**
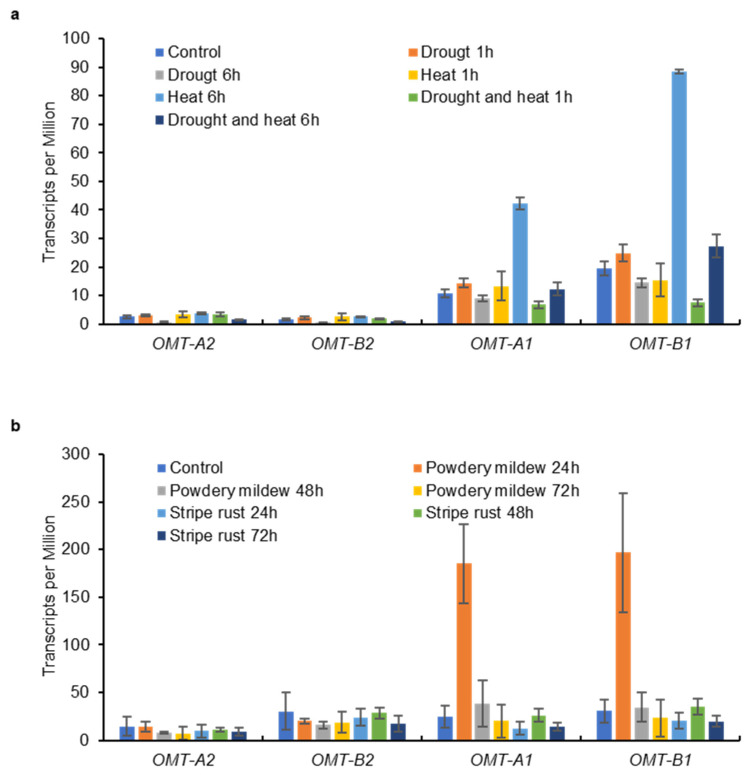
Expression levels of *OMT1* and *OMT2* homoeologs in wheat leaves subjected to abiotic (**a**) and biotic (**b**) stresses. Data on leaves of hexaploid wheat cv. TAM 107 exposed to drought and heat stresses were obtained from Liu et al., 2015 [21] and those on leaves of hexaploid wheat cv. N9134 inoculated with powdery mildew and stripe rust pathogens were obtained from Zhang et al., 2014 [22]. Transcripts per Million [means ± S.D. of two (drought and heat stresses) or three (fungal pathogens) biological replicates] are shown.

Tricin is still produced in leaves and glumes of wild-type plants where low expression of *OMT2* homoeologs was observed, suggesting that OMT2 is functional in these tissues (Figure 4 and Figure 5). However, the *omt2* mutants and TILLING control showed similar soluble phenolic metabolite profiles, including tricin, in leaves and glumes (Figure 6), suggesting that the loss of OMT2 activities had a small impact on the metabolite profile of the tissues. This could be due to additional OMT activities present in the *omt2* mutants, potentially OMT1, which can compensate for the loss of OMT2 in *omt-A2 omt-B2*. The protein sequences of OMT-A1 and OMT-A2 share 94.7% identity, and OMT-B1 and OMT-B2 are 94.4% identical, respectively (Figure 8a). In addition, the *OMT1* and *OMT2* homoeologs exhibited similar expression profiles in different tissues at various developmental stages (Figure 4 and Figure 8b,c). Furthermore, OMT1 also preferred tricetin as a substrate in enzyme assays and was able to add more than one methyl group to tricetin [18]. However, OMT1 was considered to be distinct from OMT2 because, unlike OMT2, it only had low activities towards caffeic acid, 5-hydroxyferulic acid, and flavonols [18]. Interestingly, the expression of *OMT-A1* and *OMT-B1*, but not *OMT-A2* and *OMT-B2*, was induced in wheat leaves subjected to heat stress for 6 h or infected with powdery mildew (*Blumeria graminis* f. sp. *tritici*) for 24 h (Figure 9), suggesting that OMT1 and OMT2 may play different roles in wheat under biotic and abiotic stress conditions. TILLING mutants of *OMT1* are currently being isolated to determine its function in tricin production. It will also be useful to knockout both OMT1 and OMT2 activities to discern the overlapping activities of the two OMTs and to determine whether additional OMTs, besides OMT1 and OMT2, are involved in producing tricin.

An unexpected observation is the significantly (*p* < 0.05) increased chlorogenic acid in *omt-A2 omt-B2* relative to TILLING control (Figure 6b). The *omt-A2* and *omt-B2* single mutants also showed a trend of increased accumulation of chlorogenic acid (Figure 6b). This raises the interesting possibility that chlorogenic acid may serve as a substrate for OMT2 in wheat as the lack of OMT2 activity causes a buildup of chlorogenic acid—a reaction that has not been explored previously. On the other hand, chlorogenic acid is one of the most abundant phenolic antioxidants in the human diet [19]. It will be interesting to explore the accumulation of chlorogenic acid in edible tissues of wheat for enhanced dietary chlorogenic acid using the *omt2* TILLING mutants.

While the current analysis assessed soluble phenolics [metabolites extractable in 70% (*v*/*v*) methanol] (Figure 6), it is possible that tricin produced is incorporated in lignin and not extracted with this method. However, Mäule staining of the *omt2* mutants and TILLING control plants did not reveal any apparent differences in lignin deposition in the cell walls of stems (Figure 7). Future studies that involve lignin hydrolysis and subsequent biochemical analysis will help determine if altered OMT2 activities in the mutants lead to a difference in lignin composition, e.g., changes in lignin nucleation caused by fluctuations in tricin levels.

Overall, the TILLING mutant-based genetic approach allowed exploration of OMT2 function in wheat plants and suggested the overlapping activities of OMT1 and OMT2 towards the production of methylated flavones and lignin biosynthesis. Because the current results strongly suggest compensating activities of OMT1 in the wheat *omt2* mutants, it will be useful to examine the role of tricin in wheat stress resistance using plants lacking both functional OMT1 and OMT2. In addition, overexpression of *OMT2* can be conducted in the future in biotic and abiotic stress assays. To these ends, CRISPR (Clustered Regularly Interspaced Short Palindromic Repeats)/CRISPR-associated (Cas)-based genome editing has demonstrated its utility in wheat functional genomics, providing a powerful tool to increase, decrease, or eliminate expression/function of target genes [20]. A better understanding of OMT functions and the role of tricin in wheat-environment interactions can then be applied to wheat improvement also through the genome editing approaches.

## 4. Materials and Methods

### 4.1. Plant Growth and Measurement of Plant Growth Parameters

Wheat seeds were sterilized in a solution of 1% (*w*/*v*) sodium hypochlorite and 0.1% (*v*/*v*) Triton X-100 (Sigma-Aldrich, St. Louis, MO, USA) for 15 min, followed by multiple rinses with water. The sterilized seeds were incubated at 4 °C for 1 week and then placed under a fluorescent light at room temperature for 2 days. The germinated seedlings were planted in soil and grown in a temperature-controlled greenhouse.

To determine the growth parameters of TILLING control and the *omt-A2*, *omt-B2*, and *omt-A2 omt-B2* mutants, plants were grown in a randomized arrangement in the greenhouse. Upon natural senescence (about 4 months after seed germination), the number of spikes on each plant was counted, and the height of the tallest stem was recorded from the soil line to the top of the spike (not including the awns). Spikes were separated from the straw, and the straw was dried in an oven at 60 °C for 1 week and subsequently weighed. The spikes were dried at 37 °C for 1 week and weighed separately. The pseudo-harvest index was determined via dividing spike weight by total biomass (i.e., weight of straw and spikes).

### 4.2. Identification and Molecular Characterization of omt-A2 and omt-B2 TILLING Mutants

To identify tetraploid wheat *omt-A2* and *omt-B2* mutants in the Kronos background, the TILLING mutant database was searched using *OMT-A2* and *OMT-B2* sequences from hexaploid wheat cv. CS as a query via the program Basic Local Alignment Search Tool (BLASTn). Line 3521 (*omt-A2*) was backcrossed with the parental line Kronos to reduce the level of background mutations. The backcrossed plants were self-pollinated, and progenies genotyped using CAPS markers to identify homozygous *omt-A2* plants.

To generate the *omt-A2 omt-B2* double mutant, the homozygous *omt-A2* (line 3521, BC_1_F_2_) was crossed with the homozygous *omt-B2* (line 388, M_4_). The progeny of the cross was allowed to self-pollinate and subsequently genotyped. Homozygous wild-type (*OMT-A2 OMT-B2*), single mutants (*omt-A2*, *omt-B2*), and double mutant (*omt-A2 omt-B2*) plants were identified. The polymorphic CAPS markers are *OMT-A2*, amplification with the primers 5′-AGTTTGCCCTCATGTAGTAGC-3′ (forward) and 5′- AATTAATGCATCAGATGGGAG-3′ (reverse), followed by digestion with the restriction enzyme *Bam*HI; and *OMT-B2*, amplification with the primers 5′-TGTTCAGACAGTAGTAGCATCTCC-3′ (forward) and 5′-CATCAGAGGGGTAGACTCG-3′ (reverse), followed by digestion with the restriction enzyme *Bsr*I.

To examine the expression of *OMT-A2* and *OMT-B2*, transcriptome data of wheat cv. Azhurnaya based on the publication by Ramírez-González et al. [17] were obtained from www.wheat-expression.com (accessed on 18 November 2021). Means and standard deviations of normalized read counts were calculated according to three biological replicates for each tissue type at each developmental stage and used for the preparation of bar graphs. Transcriptome data of hexaploid wheat cv. TAM 107 exposed to drought and heat stresses, and cv. N9134 inoculated with powdery mildew and stripe rust were obtained from Liu et al., 2015 [21] and Zhang et al., 2014 [22], respectively. Means and standard deviations of Transcripts per Million were calculated for 2 (drought and heat stresses) or 3 (pathogens) biological replicates and used for the preparation of bar graphs. For comparison of OMT sequences, the protein sequences were aligned with Multiple sequence comparison by Log-Expectation (MUSCLE) [23], and the alignment was graphed using the BoxShade server (https://embnet.vital-it.ch/software/BOX_form.html (accessed on 18 November 2021)).

### 4.3. Metabolite Analysis

Leaves from 4-week-old greenhouse-grown wheat plants and green glumes from spikes at approximate anthesis (plants at ~8 weeks old) were collected and immediately frozen in liquid nitrogen. The leaf and green glume tissues were ground in liquid nitrogen into a fine powder using a mortar and pestle. Approximately 100 mg of ground tissue was extracted in 350 µL of 70% (*v*/*v*) methanol with sonication for 20 min at room temperature and then centrifuged twice at 17,000× *g* for 10 min each. The supernatant recovered from each sample was transferred to an HPLC vial, and 10 µL was used for injection.

Wheat metabolite extracts were separated on a reverse-phase HPLC using a Zorbax Stablebond C_18_ column (Agilent, Santa Clara, CA, USA) following a gradient between A (0.1% formic acid in water) and B (acetonitrile) at a flow rate of 1 mL min^−1^ as described previously [24]. Commercial standards, including tricin, tricetin, apigenin, luteolin, quercetin, kaempferol, myricetin, chlorogenic acid, caffeic acid, and *trans*-cinnamic acid (all from Sigma-Aldrich), were also analyzed using the same HPLC system. Individual peaks of interest were collected from multiple HPLC runs, concentrated in a vacuum centrifuge, and subjected to MS analysis. The MS analysis was performed with a Thermo Electron LTQ-Orbitrap mass spectrometer (Thermo Scientific, Waltham, MA, USA) in the positive mode using electrospray ionization and with a flow rate of 0.2 mL min^−1^. Ten μL of sample was injected for each run, and the *m*/*z* was observed between 200 and 2000 Da.

### 4.4. Histochemical Staining of Lignin in Wheat Stem Sections

Stems of 3-month-old wheat plants were cut at the midpoint between the flag leaf node and the spike into 2–3 cm sections using a razor blade. The stem sections were embedded in 5% agarose and sliced off into 50 µm sections using a Vibratome 1000 Plus sectioning system (The Vibratome Company, St. Louis, MO, USA). The Mäule staining procedure was modified slightly from a previously established method [25]. Briefly, sectioned wheat stems were incubated in 1 mL of 0.5% (*w*/*v*) potassium permanganate (Alfa Aesar, Haverhill, MA, USA) solution for 4 min and rinsed with water. The stem sections were then incubated in 1 mL of 3.7% (*v*/*v*) hydrochloric acid (Sigma-Aldrich) for 5 min. The hydrochloric acid solution was quickly removed, and 1 mL of ammonium hydroxide (EMD Millipore, Burlington, MA, USA) was added immediately. Following removal of 500 µL of ammonium hydroxide, stem sections were pipetted onto 1 mm-thick glass slides and imaged using a RogRes CT Scan digital camera (Jenoptik, Jenna, Germany) with 10 × objective on an Olympus BH-2 compound microscope (Olympus Optical Co., Ltd., Tokyo, Japan).

### 4.5. Data Analysis

The plant growth and metabolite data were analyzed in R using Analysis of Variance (ANOVA) and Tukey’s Honestly Significant Difference (HSD) test.

## Data Availability

The data presented in this study are available in the article.

## Abbreviations

ANOVA, Analysis of Variance; BLAST, Basic Local Alignment Search Tool; Cas, CRISPR-associated; COMT, caffeic acid 3-*O*-methyltransferase; CRISPR, Clustered Regularly Interspaced Short Palindromic Repeats; HPLC, High-Performance Liquid Chromatography; HSD, Honestly Significant Difference; MS, Mass Spectrometry; MUSCLE, Multiple sequence comparison by Log-Expectation; OMT, *O*-methyltransferase; ORF, open reading frame; SAM, *S*-adenosyl-L-methionine; TILLING, Targeting Induced Local Lesions in Genomes.
